# Carry-Over Niches for Lepidopteran Maize Stemborers and Associated Parasitoids during Non-Cropping Season

**DOI:** 10.3390/insects10070191

**Published:** 2019-06-28

**Authors:** Bonoukpoè Mawuko Sokame, François Rebaudo, Boaz Musyoka, Julius Obonyo, Duna Madu Mailafiya, Bruno Pierre Le Ru, Dora Chao Kilalo, Gerald Juma, Paul-André Calatayud

**Affiliations:** 1 International Centre of Insect Physiology and Ecology (ICIPE), Nairobi P.O. Box 30772-00100, Kenya; 2 Department of Plant Science and Crop protection, University of Nairobi, Kangemi, Nairobi P.O. Box 29053 00625, Kenya; 3 UMR IRD 247 Laboratoire Evolution, Génomes, Comportement et Ecologie, Diversité, Ecologie et Evolution des Insectes Tropicaux, CNRS, 91198—Gif-sur-Yvette, France and Université de Paris-Sud, 91405 Orsay, France; 4 Department of Biochemistry, University of Nairobi, Nairobi P.O. Box 30197-00100, Kenya

**Keywords:** wild plants, maize residues, habitat and pest management, biological control

## Abstract

Sources of infestation are the key elements to be considered in the development of habitat management techniques for the control of maize stemborers. Several wild plants, grasses mostly, have been identified that serve as hosts for stemborers and their parasitoids during the off-season when maize is not present in the field. However, their abundance is much lower in wild plants compared to cultivated fields. Thus, the role of wild plants as a reservoir for cereal stemborers and their parasitoids is still controversial, particularly in agro-ecosystems with reduced wild habitat. We studied the occurrence of different maize stemborers and associated parasitoids in maize stem residues and wild grasses during non-cropping seasons as potential carry-over populations to subsequent early season maize plants. Surveys were conducted in the central region of Kenya during long and short dry seasons in maize residues and wild grasses as well as during the two rainy seasons in maize plants at earlier and late whorl stages during the years of 2017 and 2018. Wild habitat had a higher species diversity than maize residues habitat, but maize residues had a higher abundance of maize stemborer species, such as *Busseola fusca*, *Sesamia calamistis*, and *Chilo partellus*, and of associated parasitoid species (i.e., *Cotesia flavipes* and *Cotesia sesamiae*) than wild plants. Our surveys, complemented by field parasitoid releases of *C. flavipes* and *C. sesamiae*, indicated that maize residues constitute a better refugia reservoir not only of the maize stemborers but also of *C. flavipes* and *C. sesamiae* during non-cropping seasons as compared to wild plants and, thus, might constitute in this region the main source of both stemborers and *C. flavipes*/*C. sesamiae* carry-over in maize plants during the subsequent cropping season. Thus, systematic destruction of maize residues would not help the biological control of lepidopteran stemborers. This is particularly true in areas with reduced wild habitat.

## 1. Introduction

Lepidoptera stemborers, among the superfamilies of Noctuoidea and Pyraloidea, attack host plants belonging to the Poaceae, Cyperaceae, Typhaceae, and Juncaceae families with overlapping spatial and temporal distribution. Several studies report that, while more than 300 stemborer species infest wild plants, only 21 stemborer species attack cereal crops, mainly maize (*Zea mays* L.), sorghum (*Sorghum bicolor* L.), and millet (*Pennisetum glaucum* (L.) R. Br.), in various parts of Africa [1,2,3,4,5,6,7,8]. A few, i.e., the noctuids *Busseola fusca* Fuller and *Sesamia calamistis* Hampson, the crambid *Chilo partellus* Swinhoe, and the pyralid *Eldana saccharina* (Walker), are cereal crops of economic importance [9]. Yield losses vary with the region but generally range from 10% to 80% depending on infestation by the pest species and the crop growth stage [10,11].

Lepidopteran stemborers that attack maize are poly/oligo-phagous and feed on other cultivated and wild plants [12,13,14,15,16,17,18]. In sub-Saharan Africa, cereal crops are mostly grown in small fields surrounded by land that is occupied by wild plants of lepidopteran stemborers. Some poly/oligo-phagous stemborers that are found on wild plants, such as *Chilo orichalcociliellus* Strand and *Pirateolea piscator* (Fletcher), are occasionally found on cultivated cereal crops [13,19]. However, they are more frequently found on cultivated crops; for example, *Busseola segeta* (Bowden) infests 13–61% of maize fields in Western Kenya [20].

In Kenya, the larvae of *B. fusca* (an African maize stalk borer), *S. calamistis* (a pink stem borer), and *C. partellus* (a spotted stem borer) contribute up to 82% of maize yield losses [9,21,22,23]. In the context of a biological control program, the most commonly used parasitoids are the larval parasitoids, including *Cotesia flavipes* Cameron and *Cotesia sesamiae* (Cameron) (Hymenoptera: Braconidae), followed by the pupal parasitoids *Xanthopimpla stemmator* Thunberg (Hymenoptera: Ichneumonidae) and *Pediobius furvus* Gahan (Hymenoptera: Eulophidae), and then the tachinid *Siphona* sp. [19,24]. Among these natural enemies, the larval parasitoids *C. flavipes* and *C. sesamiae* have been found to be the most efficient to control lepidopteran stemborers and, for example, *C. flavipes* was used in a classical biological control program against *C. partellus* in East Africa [24,25]. *Cotesia sesamiae* efficiently parasitized *S. calamistis* and *B. fusca* larvae [9]. During cropping seasons, the maize lepidopteran stemborers *B. fusca*, *S. calamistis*, and *C. partellus* and their associated parasitoids *C. flavipes* and *C. sesamiae* are more abundant in maize fields as compared to wild plants [26,27,28,29]; and perennation (the ability to survive from one cropping season to the next) by both *C. flavipes* and *C. sesamiae* occurs mainly in cultivated habitats [28,29]. This supposes that the parasitoids follow their lepidopteran maize stemborer hosts during non-cropping seasons, either in larvae feeding on wild plants surrounding maize fields [13,17,18,30] or in diapausing larvae in maize residues left in the maize field after harvest [13,31,32,33,34,35]. However, since the abundance of both lepidopteran stemborers and their parasitoids is much lower in wild plants compared to maize plants [26,29], the role of wild plants as a reservoir for cereal stemborers and their parasitoids is still controversial, particularly in agro-ecosystems with reduced wild habitat.

In this context, the objectives of the present study were to (i) compare the diversity and abundance of stemborers and their associated parasitoids between maize residues and wild plants identified as hosts for maize stemborers [2,3] during non-cropping seasons; (ii) study the relationships between maize stemborer species and their respective parasitoid abundances in either maize residues or wild plants during non-cropping seasons and in maize plants during subsequent cropping seasons; and (iii) determine the main potential niches that could harbour parasitoids after releasing the larval parasitoids *C. flavipes* and *C. sesamiae* in the studied fields.

## 2. Materials and Methods

### 2.1. Study Localities and Sampling Design

Surveys on maize residues and alternative wild plants were conducted in two different localities (Makutano and Murang’a) situated in the central region of Kenya. These two localities are characterized by a reduced wild habitat. Only three or four wild grass species surrounded the maize fields in Murang’a and Makutano, respectively, during our field surveys (see results of Table 2), as compared to the Kitale, Mtito Andei, Kakamega, and Muhaka agroecological zones studied by Mailafiya [12], where many wild plants surround the maize fields (see Supplementary Table S1) and small cultivated fields dominate [36]. Makutano (0°43′37′’ S, 37°16′22′’ E; 1150–12,250 m above sea level (a.s.l.)) and Murang’a (0°55′23′’ S, 37°09′00′’ E; 1267–1500 m a.s.l) have a bimodal rainfall distribution, two cropping seasons (from April to June and from October to December), a mean annual precipitation of 981 mm and 1195 mm, and temperature ranges of 12.05–26.04 °C and 11.01–22.7 °C, respectively. Different areas are dominated by different stemborer pest species [37,38]. In the Makutano area, *C. partellus* and *S. calamistis* co-infest maize fields [38]. In the Murang’a area, *B. fusca* and *S. calamistis* co-exist [38]. These areas have, therefore, been chosen for the study, so as to cover these three stemborer species that are economically important pests for maize in Kenya. Similarly, in the agroecological zones studied by Mailafiya [12], *C. partellus* and *S. calamistis* were found to co-infest maize fields at Mtito Andei and Muhaka, which are situated in the southeasthern region of Kenya, while *B. fusca* and *S. calamistis* were found to co-exist at Kitale and Kakamega in Western Kenya. Maize is the predominant crop in all areas and is grown each wet season.

The surveys were done for two years (2017 and 2018). Three farmers’ fields were randomly selected in each locality. Similarly to [12], maize residues in each field and adjacent wild habitat were sampled two to three times during each non-cropping season of each year. Maize plants were sampled two times in maize fields during each subsequent cropping season of each year. A total of 10 surveys were conducted in each farmer’s field in maize residues and adjacent wild plants during the non-cropping seasons and eight surveys were conducted in each farmer’s field in maize plants during the cropping seasons of the two years of the survey (Table 1).

### 2.2. Data Collection

#### 2.2.1. Sampling for the Diversity and Abundance of Lepidopteran Stemborers and Associated Larval and Pupal Parasitoids

##### In Maize Residues during Non-Cropping Seasons

The optimal number of maize residues sampled in each field during each survey was determined using the equation described by Zar [39]:

(1) n=14Dd2(Zα22)

where Zα22 is the standard normal deviate (1.96), *d* is the permitted error (0.1) resulting in a uniform number of maize residues in all farms, and *D* is the design effect (1).

(2) $$n=\frac{1}{4\times 1\times 0.1^{2}}\left(1.96^{2}\right)=96.04\approx 100$$

Hence, 100 maize residues were randomly sampled in each field during each survey. Each residue sampled was inspected and dissected for stemborer infestations, and stemborer larvae and pupae were recovered from each maize residue. Stemborer larvae and pupae were counted, placed in glass vials (8.5 × 2.7 cm), and reared in the laboratory to confirm species identification at the adult stage or to recover the parasitoids as follows.

The collected stemborer larvae were reared on an artificial diet developed by Onyango and Ochieng‘-Odero [40] in cylindrical glass vials (8.5 × 2.5 cm) plugged with cotton wool and kept under ambient conditions in the laboratory (25 ± 1 °C; 67 ± 4% relative humidity) until pupation or cocoon formation in case of parasitism. Pupae taken out of the artificial diet/maize stems were sexed readily by examining the external morphology of the ventral surface of the eighth and ninth abdominal segments using a Wild dissecting microscope according to the method described by Underwood [41]. Then, they were kept in separate plastic containers (16 × 10 cm) closed with perforated plastic lids until adult emergence for stemborer species identification. Parasitoid cocoons and puparia collected from stemborer larvae/pupae were separately kept in glass vials (2.5 cm in diameter and 7.5 cm in height) until adult emergence and conserved in 70% ethanol for species identification [42,43] in collaboration with the biosystematics unit of the International Centre of Insect Physiology and Ecology (ICIPE).

##### In Wild Plants during Non-Cropping Seasons

To estimate stemborer diversities and densities on wild plants, 50–100 plants/tillers were randomly sampled per plant species in each field depending on the availability of plant species. Each plant/tiller selected was dissected in the field for recovery of larvae and/or pupae. They were brought into the laboratory for both stemborer and parasitoid (in case of parasitism) species identification using the above-described protocol.

##### In Maize Plants during Cropping Seasons

The farmers’ maize fields that were sampled in each locality were each approximately 0.5–1.5 ha in size. The sampling followed the procedure described by Overholt et al. [44]. Each sampled maize field, at early and late whorl stages, i.e., with plants between 4 and 8 weeks old, respectively (plant stages that are succeptible to stemborer attack), was divided into four quadrants, and 25 plants were randomly selected from each quadrant following a zig-zag pattern, giving a total of 100 plants per field, according to the equation described by Zar [39]. Plants with stemborer damage symptoms, such as scarified leaves (window panes and pin holes), frass, dry leaves and shoots (dead hearts), or a bored (entrance or exit) hole, were uprooted from the field and dissected for recovery of stemborer larvae and/or pupae from the stems or whorls. All collected stemborer larvae and pupae were counted, then brought into the laboratory for both stemborer and parasitoid species identification using the above-described protocol.

#### 2.2.2. Releases of *Cotesia flavipes* and *Cotesia sesamiae* in the Studied Farmer’s Fields

The purpose of this experiment was to identify the refugia habitat (maize residues or wild plants) of parasitoids associated with maize stemborers during the non-cropping season. For that, the larval parasitoids *C. flavipes* and *C. sesamiae* were used for releases to “boost” the abundance of these parasitoids in the studied fields. For each parasitoid species, adults, cocoon masses, and parasitized larvae were released (Table 1). These different stages were released to enhance the likelihood of the establishment of the parasitoid, as previously done by ICIPE in a biological control program using *C. flavipes* towards *C. partellus* in East and Southern Africa [45]. In each cultivated field of maize plant, the parasitoids were released in the center of the field to allow the parasitoids to find the host.

Cocoon masses of *C. flavipes* and *C. sesamiae* were obtained from the Animal Rearing and Containment Unit (ARCU) at ICIPE Duduville, Nairobi, Kenya. When parasitoids were ready to emerge from cocoons, they were placed in a large sleeve cage (35 cm^3^) until emergence. After emergence, a 20% honey/distilled water solution offered on cotton wool in a Petri dish was introduced into the cage to provide food. The cage was then placed under incandescent light for ca. 24 h to stimulate mating. The hosts, larvae of *C. partellus* for *C. flavipes* and larvae of *S. calamistis* for *C. sesamiae*, were obtained from colonies reared at ARCU. These hosts were reared on an artificial diet up to the fourth instar. They were removed from the artificial diet and transferred to maize stems, where they were permitted to feed for 24 h. Then, they were exposed to parasitoids using the hand stinging method [46]. After oviposition, the larva was immediately removed from the cage to avoid super-parasitism. The parasitized larvae were placed into small vials (7.5 × 2.5 cm) containing artificial diet and incubated at 25 °C until cocoon formation 10–15 days after exposure. The cocoon masses were removed from the artificial diet, placed in a clean vial, and held until they darkened (15–20 days after exposure). At emergence, the females were allowed to mate for 24 h, after which they were ready for release.

The natural parasitism of maize stemborers by both *C. flavipes* and *C. sesamiae* in the Makutano and Murang’a areas was initially very low, even nil. The plots were surveyed in these localities before parasitoid release to confirm the scarcity of parasitism by both *C. flavipes* and *C. sesamiae* (see results of Figure 4). Since *C. flavipes* was used in a classical biological control program against *C. partellus* [24,25] and *C. sesamiae* was found to efficiently parasitize *S. calamistis* and *B. fusca* larvae [9], *C. flavipes* was released in the Makutano area where *C. partellus* and *S. calamistis* co-infest maize fields [38], whereas *C. sesamiae* was released in the Murang’a area where *B. fusca* and *S. calamistis* co-exist [38]. Releases were done at the maize whorl stage, which is the favored stage for oviposition by stemborers. Adults and cocoon masses were released in the whorl of infested plants, while parasitized larvae were placed inside the leaf sheaths. The releases took place in December 2017 and in June 2018 in three maize fields per locality (Table 1) early in the morning or late in the afternoon to allow the parasitoids to become acclimated and locate refuges before the temperature became too high.

Thereafter, the parasitism rates after the parasitoid releases in stemborers found in either maize residues or wild plants during non-cropping seasons and the parasitism rates in stemborers found in maize plants during the subsequent cropping seasons were estimated using the species identification protocol and the collected parasitized stemborer larvae.

### 2.3. Data Analysis

All analyses were carried out in the R software version 3.5.1 (R Core Team, Vienna, Austria) [47]. The BioFTF R package [48], considering both the richness and the evenness, was used to compare the diversity of maize stemborer communities and their associated parasitoids in maize residues and wild plants. It exploits the β diversity profile model (Equation (3)):

(3) $$\Delta \beta =\overset{s}{\underset{i=1}{\sum }}\frac{\left(1-p_{i}^{\beta }\right)}{\beta }p_{i};\;\beta \geq \;-1$$

For Equation (3), β = −1 generates the richness index, lim β→ 0 represents the Shannon diversity index, and β = 1 returns the Simpson Index.

The number of larvae and pupae from the 100 sampled stems/tillers/plants were compared between habitats (maize residues and wild plants) using a generalized linear model with a negative binomial error distribution (GLM.nb) due to the nature of the count data of this parameter. Significant differences were separated by Tukey’s multiple comparisons tests performed using the R package “lsmeans” [49]. From the GLM results, the Odds Ratio (O.R.) with a 95% confidence level interval (O.R. (95% CI)) was calculated.

The stemborer and associated parasitoid species composition in either maize residues or wild plants and maize plants in cultivated field habitats were pairwise compared by calculating the Morista–Horn index (C*mH*) [50]:

(4) $$C_{mH}=\frac{2\times \sum \left(n_{ia}\times n_{ib}\right)}{\left(d_{a}+d_{b}\right)N_{a}\times N_{b}}$$

where *N*_a_ and *N*_b_ are the total number of individuals in maize residues and maize plants in cultivated fields or in wild plants and maize plants in cultivated fields, respectively; and *n*_ia_ and *n*_ib_ are the number of individuals of a given species *i* in maize residues and maize plants in cultivated fields or in wild plants and maize plants in cultivated fields, respectively:

(5) $$d_{a}=\frac{\sum n_{ia}^{2}}{N_{a}^{2}}$$

and

(6) $$d_{b}=\frac{\sum n_{ib}^{2}}{N_{b}^{2}}$$

high values of C*mH* indicate increasing similarity between the two habitats, with a maximum of 1.

To establish whether the abundance of stemborer species in maize plants of cultivated fields was correlated to those either in maize residues or wild plants during the non-cropping season, we performed a principal component analysis (PCA) using two R packages called “FactoMineR” and “Factoextra” [51]. To elucidate correlations between the abundance of each species in maize plants of cultivated fields and those in either maize residues or wild plants, we constructed a correlogram (R package “corrplot ”) and performed a correlation test using the Pearson method. Furthermore, the proportions of females and males of each species (*B. fusca*, *S. calamistis*, and *C. partellus*) in maize residues and maize plants in cultivated fields were evaluated and compared using the two-tailed Fisher’s exact probability 2 × 2 test (http://graphpad.com/quickcalcs/contingency2/).

Parasitoids recovered in maize residues or wild plants during non-cropping seasons as well as in maize plants of cultivated fields during the subsequent cropping seasons in Makutano and Murang’a before (pre-release) and after or during *Cotesia flavipes* and *Cotesia sesamiae* release (post and during release) are expressed as mean (± standard error (SE)) of parasitized hosts recorded per field. The parasitism rate for each parasitoid for the *B. fusca*, *S. calamistis*, and *C. partellus* species was quantified as the proportion of parasitized larvae among the total number of the given species in each habitat (maize residues, wild plants, and maize plants). Parasitism rates between habitats were compared using a proportion test.

## 3. Results

### 3.1. Diversity and Abundance of Lepidopteran Stemborers and Associated Parasitoids in Maize Residues and Wild Plants during Non-Cropping Seasons

A total of 785 stemborers were collected, of which 653 were obtained from maize residues and 132 from wild plants. Among them were five Noctuidae, two Crambidae, and one Pyralidae (Table 2).

In each locality, the wild plant habitat had a higher stemborer species diversity than the maize residue habitat (Table 3).

**Figure 1:**
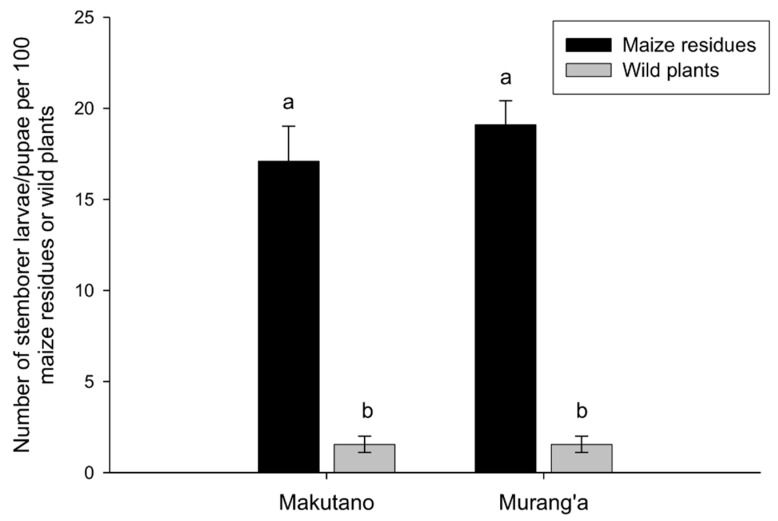
Number of stemborers of *Busseola fusca*, *Sesamia calamistis*, and *Chilo partellus* found in maize residues and wild plants per 100 maize residues or wild plants sampled in two different localities in the central region of Kenya (Makutano and Murang’a) during non-cropping seasons in 2017 and 2018. Non-significant differences between maize residues versus wild plants are shown by identical letters determined using Tukey’s multiple comparisons tests with the R package “lsmeans”, following a generalized linear model (GLM) with a negative binomial error distribution.

However, wild plants had a lower stemborer abundance than maize residues, from which only *B. fusca*, *S. calamistis*, and *C. partellus* were obtained (Figure 1; GLM.nb results: for Makutano (O.R. = 0.09 (0.06–0.14), *p* < 0.00010) and for Murang’a (O.R. = 0.08 (0.05–0.11), *p* < 0.0001)).

During the two years of surveys, the parasitoid species that were obtained were from the families of Braconidae, Ichneumonidae, Eulophidae, and Tachinidae (Table 4). Among them, the braconids *C. flavipes* and *C. sesamiae* were the most abundant and widespread species after release (see Figure 5); they were mostly obtained from *B. fusca*, *S. calamistis*, and *C. partellus* inhabiting maize residues.

Similarly to the stemborer species, the wild plant habitat had a higher parasitoid species diversity than the maize residue habitat (Table 5).

### 3.2. Relationships in Insect Abundance between Maize Stemborer Species and Their Respective Parasitoids in Either Maize Residues or Wild Plants during Non-Cropping Seasons and in Maize Plants in Cultivated Fields during Subsequent Cropping Seasons

During subsequent cropping seasons, a total of 1200 stemborer larvae and pupae consisting of *B. fusca*, *S. calamistis*, and *C. partellus* were collected in maize plants in the two localities (Table 6). The average number per sample was 11.87 ± 6.51 and 9.00 ± 4.51 for *C. partellus* and *S. calamistis*, respectively, with an overall average of 10.72 ± 3.94 in Makutano. The average number per sample was 20.95 ± 7.76 and 7.58 ± 2.98 for *B. fusca* and *S. calamistis*, respectively, with an overall average of 14.21 ± 4.51 in Murang’a.

In each locality, the Morista–Horn indices were higher between maize plants versus maize residues than in comparisons between maize plants versus wild plants (Table 7).

**Figure 2:**
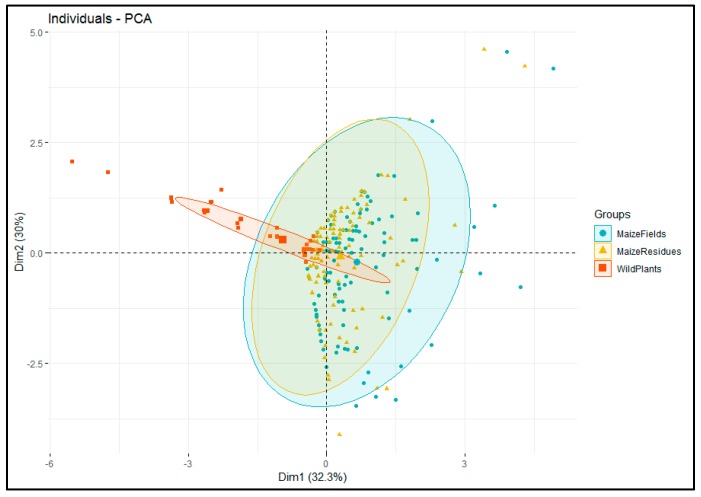
The principal component biplot showing the relation between abundance of stemborer species in maize plants of cultivated fields during cropping season and those in either maize residues or wild plants during non-cropping season.

The Principal Component Analysis (PCA), which was performed to correlate the abundance of stemborer species between habitats (maize residues, wild plants, and maize plants), revealed that stemborer species abundance in cultivated maize fields was highly correlated to abundance in maize residues, yet not to stemborer abundance in wild grasses (Figure 2).

**Figure 3:**
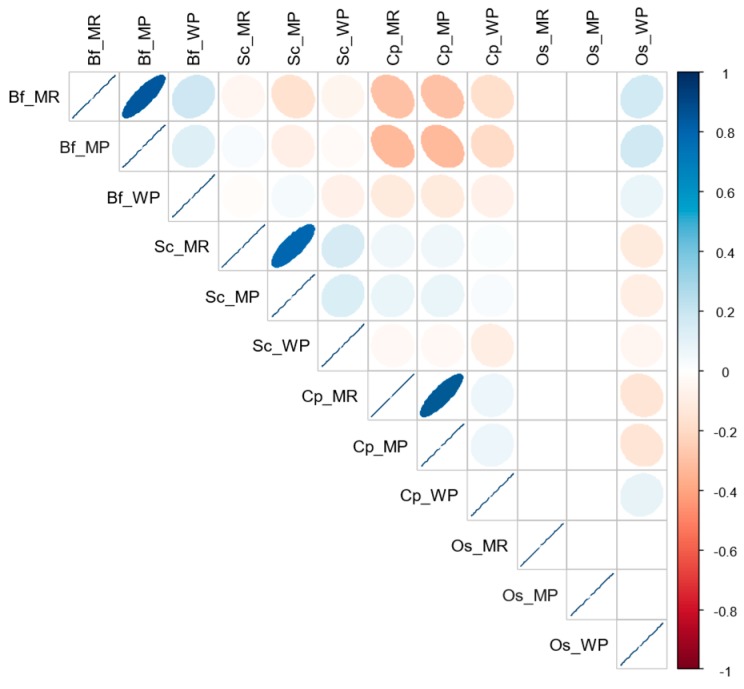
A correlogram highlighting the direction and intensity of the correlation between the abundance of each stemborer species in maize plants of cultivated fields during cropping season and those in either maize residues or wild plants during non-cropping season. The blue color denotes a positive correlation and the red color a negative correlation. Higher intensity of the color indicates a strong correlation. Bf_MR, *B. fusca* in maize residues; Bf_MP, *B. fusca* in maize plants; Bf_WP, *B. fusca* in wild plants; Sc_MR, *S. calamistis* in maize residues; Sc_MP, *S. calamistis* in maize plants; Sc_WP, *S. calamistis* in wild plants; Cp_MR, *C. partellus* in maize residues; Cp_MP, *C. partellus* in maize plants; Cp_WP, *C. partellus* in wild plants; Os_MR, other species in maize residues; Os_MP, other species in maize plants; Os_WP, other species in wild plants. For other species (Os), see Table 2.

The correlations between the abundance of each stemborer species in maize plants of cultivated fields and those in either maize residues or wild plants are clearly illustrated in the correlogram shown in Figure 3. The abundance of *Busseola fusca, Sesamia salamistis*, and *Chilo partellus* in maize plants of cultivated fields were significantly and positively correlated with their carry-over abundances in maize residues during non-cropping seasons (*r* = 0.84, *t* = 17.00, *df* = 118, *p* < 0.0001; *r* = 79, *t* = 14.06, *df* = 118, *p* < 0.0001; *r* = 0.96, *t* = 28.54, *df* = 118, *p* < 0.0001, respectively), while no relationship was evidenced between their abundances in wild plants (*r* = 0.12, *t* = 1.34, *df* = 118, *p* = 0.18; *r* = 13, *t* = 1.45, *df* = 118, *p* = 0.14; *r* = 0.06, *t* = 0.68, *df* = 118, *p* = 0.49, respectively).

**Figure 4:**
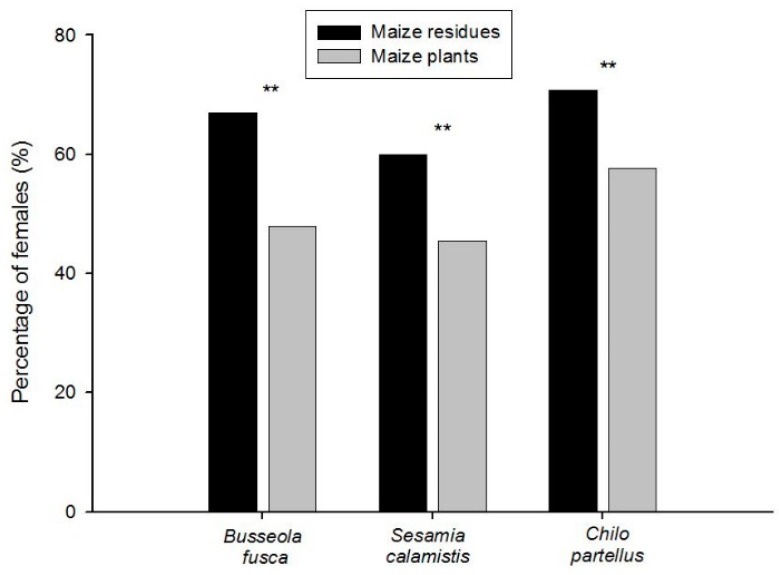
Percentages of females of *Busseola fusca*, *Sesamia calamistis*, and *Chilo partellus* found in the maize residue habitat during the non-cropping seasons and on maize plants in the cultivated habitat during the subsequent cropping seasons. The proportion of males and females was set to 100% to calculate the percentage of females. The proportions of males and females were compared between habitats for each species using a two-tailed Fisher’s exact probability 2 × 2 test (**: *p* < 0.01).

Furthermore, for each species, the percentage of females was significantly higher in the maize residue habitat than in maize plants of the cultivated habitat (Figure 4; *B. fusca*: *p* = 0.0002; *S. calamistis*: *p* = 0.001; and *C. partellus*: *p* = 0.005; two-tailed Fisher’s exact test).

**Figure 5:**
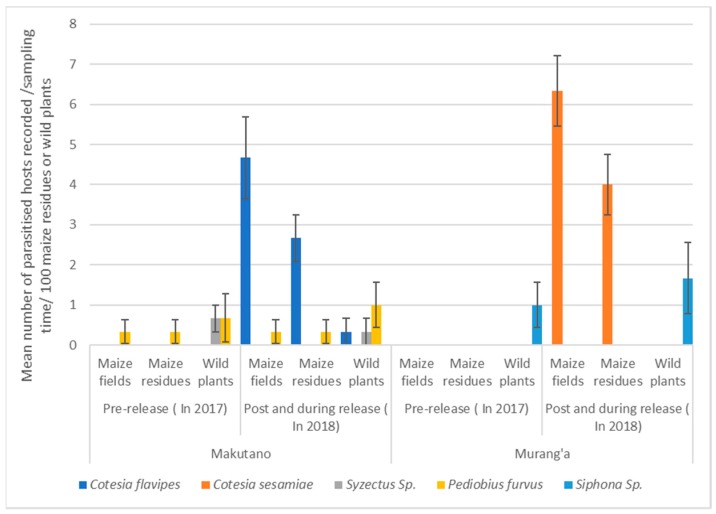
The mean number (±SE) of parasitoid species recovered per field in maize residues or wild plants during non-cropping seasons as well as in maize plants of cultivated fields during the subsequent cropping seasons in Makutano and Murang’a before (pre-release) and after or during (post and during release) *Cotesia flavipes* and *Cotesia sesamiae* releases.

Before parasitoid release, *C. flavipes* or *C. sesamiae* were not recovered from either maize plants, maize residues, or wild plants in Makutano and Murang’a (Figure 5). Three parasitoid species (*Syzectus* sp., *Pediobius furvus*, and *Siphona* sp.) were recorded, mostly in wild plants, of which only *Pediobius furvus* was present in maize residues and maize fields (Figure 5). After and during the releases, *C. flavipes* and *C. sesamiae* were recovered mostly from maize plants during subsequent cropping seasons and maize residues during non-cropping seasons (Figure 5).

The Morista–Horn similarity indices that were calculated for parasitoid abundances were higher in the comparison between maize plants versus maize residues than in the comparison between maize plants versus wild plants (Table 7).

The parasitism rates of *C. flavipes* and *C. sesamiae* that were obtained in the respective host species did not differ between habitats. These results, summarized in Table 8, show no difference in parasitism rate between the different habitats. Overall, the parasitism levels were low, and generally nil in wild habitats.

## 4. Discussion

Although a higher diversity of stemborers and parasitoids in wild habitats as compared to cultivated habitats has already been well-reported in the literature [2,3,7,19,26,27,28,29,52,53,54,55,56,57], this study highlights for the first time a broader host range in wild habitats as compared to maize residue habitats. This variation in distribution amongst insect diversity between wild and cultivated habitats might be a consequence of anthropogenic changes in the ecology of the availability of food resources, constraints of natural enemies, and the evolution of competitive interactions [58,59,60]. Diniz et al. [61], who studied species richness of flower-head insects (Tephritidae: Diptera) in natural and cultivated habitats, have mentioned that anthropogenic alterations in the landscape determine the impoverishment of insect diversity in cultivated habitats. For stemborers’ parasitoids, Mailafiya et al. [28] showed that parasitoid diversity was lower in locations where maize cultivation was practiced on a commercial scale and where intense grazing activities persist across seasons. Nevertheless, a higher insect diversity does not mean a higher insect abundance. In fact, the abundance of stemborers, which are pest of maize plants (i.e., *B. fusca*, *S. calamistis*, and *C. partellus*) and their associated parasitoids (i.e., *C. flavipes* and *C. sesamiae*), was found to be higher in the maize residue habitat as compared to the wild plant habitat. It was previously found that both stemborers and parasitoids associated with pest stemborers are generally less abundant in wild habitats than in maize plants of cultivated habitats [26,29,52]. Although natural habitats surrounding cereal crops serve as refugia for sustaining the diversity of both stemborers and parasitoids from adjacent cereal fields [12,19], the abundance of stemborers and associated parasitoids is very low in the wild as compared to cultivated fields [26,61]. A possible explanation is the low abundance of wild plant species surrounding our studied fields. In fact, when analyzing the data obtained by Mailafiya [12] in other agro-ecological zones in Kenya, the correlation between the abundance of stemborers in the maize fields and in maize residues was greater for maize fields surrounded by a low diversity of wild plants (Kitale and Mtito Andei) than for those surrounded by a high diversity of wild plants (Kakamega and Muhaka) (see Tables S1 and S2 and Figures S1 and S2). In addition, these findings indicate that this correlation of the stemborer species abundance in maize plants of cultivated fields with those in maize residues depend not only on the abundance of wild plant species in the agro-ecosystem but also on the abundance of wild plants that are suitable to maize stemborers, such as *Megathyrsus maximus* and *Sorghum arundicaneum* (wild sorghum), surrounding maize fields. Another possible explanation is the generally higher survival and growth rates of the stemborers, and, thus, their associated parasitoids, on cultivated plants as compared to wild plants [52,62,63,64,65,66,67].

Overall, the role of wild plants surrounding cultivated areas in the carry-over of stemborer pests and their associated parasitoids during the non-cropping seasons is limited, suggesting that other niches, such as maize residues, might be also involved. It has been well-reported that maize residues left in the maize field after harvest constitute an important source of the maize pest stemborers that are involved in the carry-over of the insect pests on maize plants for the subsequent cropping season [13,14,15,31,32,33,34,35,68].

In our study, the highest Morista–Horn similarity indexes of both maize stemborers and their associated parasitoids obtained in maize plants of the cultivated habitat with maize residues as compared with the wild habitat indicate that maize residues might constitute an important refugia source not only of the maize stemborers but also of their associated parasitoids. Analyzing the data on stemborer and parasitoid recoveries between wild and maize residue habitats during non-cropping seasons that were obtained by Mailafiya [12] in Kitale and Mtito Andei and Kakamega and Muhaga confirm our results (see Tables S3 and S4). It was even reported that *S. calamistis* populations living in wild habitats differ from those living in cultivated habitats [69], which compromises wild habitats as a refugia source of that species coming from maize plants in cultivated habitats. Although it was shown that *B. fusca* infestation might originate from specimens coming from outside the maize fields, probably from quite a distance [70], based on the insect abundance relationships between maize residues and maize plants, we cannot preclude maize residues from being the main reservoir of maize stemborers, particularly when wild plants surrounding maize fields are scarce. It is well-reported that *B. fusca,* for example, survive the dry season as larvae diapausing into maize residues left in the field after harvest [14,15,31,32,34,68]. The high positive correlation between the abundance of stemborers in the maize fields and in maize residues obtained in our study reinforced the fact that maize residues might serve as the main reservoir of maize stemborers and, thus, as the main source of the carry-over of the maize pest for the next cropping season.

In addition, for each species, stemborers recovered from maize residues gave rise to a significantly greater percentage of females as compared to stemborers recovered in maize plants. This is in accordance with Gebre-Amlak [71], who reported that the first generation of *B. fusca* coming from diapause larvae found earlier in the cropping season gave more females than males compared to further generations. These seasonally dependent sex ratio variations might be due to either climatic and environmental factors or intrinsic factors of the insect to ensure the perennity of its species by a female-biased sex ratio distortion when the conditions became unfavorable. Kageyama et al. [72], studying the occurrence of feminizing bacteria in an insect by a female-biased sex ratio in *Ostrinia furnacalis* (Lepidoptera: Crambidae), the Asian corn borer, concluded that, in the sex determination systems in lepidopteran insects, chromosomal males are feminized by a cytoplasmic agent, most probably parasitic bacteria, according to the conditions. This phenomenon was confirmed in other insect species [73,74,75,76,77] and might be explored in lepidopteran stemborers in relation to the habitat.

In addition, the absence of both *C. flavipes* and *C. sesamiae* in the field before any release could be due to: (i) the frequent use of pesticides; (ii) the systematic use of maize residues for animal feed during dry seasons; or (iii) the climate change adaptation of both parasitoids and hosts to dry seasons, which, in the last five years, have been particularly long [36]. In contrast, after and during the releases, although the parasitism rates were low (which normally occurs after the first parasitoid release [45] and according to the season, the year, and the locality [78,79]), the highest recovery of *C. flavipes* and *C. sesamiae* obtained in maize fields during the subsequent cropping seasons indicate a possible establishment of the parasitoids released in these areas. The successful establishment of *C. flavipes* after release has already been observed in different countries, including coastal Kenya [24,26,45,55,80]. In addition, the fact that, similarly to maize stemborers, *C. flavipes* and *C. sesamiae* were mostly recovered after and during the releases in maize residues during the non-cropping seasons confirmed those residues as being the main reservoir of maize stemborer parasitoids during dry periods. This aspect is important to consider in the context of biological control. In fact, it has been recommended that the maize residues (observed to be an important reservoir of maize stemborers) be burned [14,15,31,32,66] in order to diminish the risk of maize infestation by stemborers for the subsequent cropping seasons [5]. This management measure should not be adopted in areas where *C. flavipes* and *C. sesamiae* have been released or where the wild habitat has been drastically reduced. In those contexts, maize residues might ensure the perennity of the parasitoids during dry seasons. Considering that: (i) *C. flavipes* and *C. sesamiae* were found to be rare or absent in all habitats prior to release; (ii) parasitism by *C. flavipes* is generally low or absent in the years after biological control release [79]; and (iii) maize residues are also the main reservoir of parasitoids during dry periods [12] (see Tables S2 and S4), maize residues may also represent the main sources of stemborer parasitoids during non-cropping seasons. This suggests that maintaining residues will promote the parasitism of stemborers. However, to determine whether a buildup of the parasitoid population might occur over time, a further study might be conducted on the comparable effect of infestation and parasitism over a longer time period to confirm the influence of maize residues on both infestation and parasitoid presence. In addition, some wild plants, such as the wild sorghum *S. arundinaceum*, support a high survivorship of parasitized stemborers and, therefore, a relatively high performance of their larval parasitoids [64]. The maintenance of these wild plants is also vital for the survival and, thus, the perenity of *C. flavipes* and *C. sesamiae* in the field.

## 5. Conclusions

This study highlighted the importance of maize residue habitats as an important reservoir source of maize stemborers and their associated parasitoids that ensures the perennity of the maize stemborer parasitoids in the field during dry periods.

## Figures and Tables

**Table 1:** The number of surveys in each habitat and releases of parasitoid in maize fields at Makutano and Murang’a. The parasitoid releases were done by releasing adults, cocoon masses, or parasitized larvae.

Localities	Sampling/Release Sites	Number of Surveys in Each Habitat			Parasitoid Species Released	No. Adults	No. Cocoon Masses	No. Parasitized Larvae
		Maize Residues	Wild Plants	Maize Plants				
					2017			
Makutano	Field 1	6	6	4	*Cotesia flavipes*	500	20	8
	Field 2	6	6	4		400	20	28
	Field 3	6	6	4		400	-	10
	Total	18	18	12	*Cotesia flavipes*	1100	40	46
Murang’a	Field 1	6	6	4	*Cotesia sesamiae*	300	100	50
	Field 2	6	6	4		300	30	14
	Field 3	6	6	4		400	3	-
	Total	18	18	12	*Cotesia sesamiae*	1000	133	64
					2018			
Makutano	Field 1	4	4	4	*Cotesia flavipes*	400	40	-
	Field 2	4	4	4		200	20	-
	Field 3	4	4	4		200	20	-
	Total	12	12	12	*Cotesia flavipes*	800	80	-
Murang’a	Field 1	4	4	4	*Cotesia sesamiae*	100	20	-
	Field 2	4	4	4		100	10	-
	Field 3	4	4	4		50	5	-
	Total	12	12	12	*Cotesia sesamiae*	250	35	-

-: No release of that parasitoid stage in that field has been done.

**Table 2:** Stemborer species composition and total abundance in maize residues and wild plants during non-cropping seasons in two different localities in the central region of Kenya in 2017 and 2018. Values in parenthesis express the number of given species per 100 maize residues/wild plants sampled (mean ± standard error (SE)).

Habitats	Total Number	Stemborer Species Composition			
		Bf	Sc	Cp	Os
	Makutano				
Maize residues	290	-	202 (6.73 ± 2.16)	88 (2.93 ± 0.89)	-
Wild plants	99	-	20 (0.67 ± 0.49)	15 (0.50 ± 0.32)	61 (2.03 ± 1.50)
*Sorghum arundinaceum* *	25	-	4	8	9 Csp, 4 Mni
*Pennisetum purpureum* *	39	-	3	7	29 Csp
*Megathyrsus maximum* *	22	-	7	-	15 Mn
Cyperus sp. †	13	-	6	-	7 Mn
	Murang’a				
Maize residues	363	226 (7.53 ± 2.87)	137 (4.57 ± 1.37)	-	-
Wild plants	33	2 (0.06 ± 0.03)	11 (0.36 ± 0.28)	-	20 (0.67 ± 0.37)
*Pennisetum purpureum* *	14		9	-	5 Sn
Megathyrsus maximum *	6	2	1	-	3 Mn
*Cynodon dactylon* *	13	-	1	-	7 Ssp, 5 Mn

Os, Other species. Noctuidae (Bf, *Busseola fusca;* Mn, *Manga nubifera*; Sc*, Sesamia calamistis*; Sn, *Sciomesa nyei*; Ssp, *Sciomesa* sp.)/Crambidae (Cp, *Chilo partellus*; Csp, *Chilo* sp.)/Pyralidae (Mni, *Mussidia nigrivenella*). Plant family: * Poaceae, † Cyperaceae. -: species was absent.

**Table 3:** The stemborer species diversity ranking in maize residue and wild plant habitats during non-cropping seasons in two different localities in the central region of Kenya.

Habitats	Makutano			Murang’a		
	Richness	Shannon	Simpson	Richness	Shannon	Simpson
Maize residues	2	0.627768	0.4227348	2	0.6760856	0.4699436
Wild plants	5	1.503721	0.7378839	5	1.5600483	0.7584940

A high number indicates great diversity in the habitat.

**Table 4:** Parasitoid composition and abundance from stemborer species in maize residues and wild plants during non-cropping seasons in two different localities in the central region of Kenya in 2017 and 2018 (i.e., before and after/during parasitoid release).

Parasitoid Species	Stem Borer Species	Stem Borer Stages	Wild Plants	Total Number of Parasitized Hosts	Makutano		Murang’a	
					Maize Residues	Wild Plants	Maize Residues	Wild Plants
Hymenoptera: Braconidae								
*Cotesia flavipes*	Cp, Sc	larva	Sa	9	8	1	-	-
*Cotesia sesamiae*	Bf, Sc	larva	-	12	-	-	12	-
Hymenoptera: Ichneumonidae								
*Syzectus sp.*	-	pupa	Sa, Pm	3	-	3	-	-
Hymenoptera: Eulophidae								
*Pediobius furvus*	Cp, Sc	pupa	Sa	4	1	1	-	2
Diptera: Tachinidae								
*Siphona* (Meigen) *sp.*	Bf, Mn	larva	Pm	3	-	-	-	3

Stemborer species: Bf, *Busseola fusca*; Sc, *Sesamia calamistis*; Cp, *Chilo partellus*; Mn, *Manga nubifera*. Plant species: Sa, *Sorghum arundinaceum*; Pm, *Panicum maximum*. -: absent.

**Table 5:** Stemborer-associated parasitoid species diversity ranking of maize residue and wild plant habitats during non-cropping seasons in two different localities in the central region of Kenya.

Habitats	Makutano			Murang’a		
	Richness	Shannon	Simpson	Richness	Shannon	Simpson
Maize residues	2	0.3638339	0.1975309	1	0.2852830	0.1420118
Wild plants	3	0.9824805	0.5600000	2	0.6861506	0.4800000

A high number indicates great biodiversity in the habitat.

**Table 6:** Total number of larvae/pupae of lepidopteran maize stemborer species (with the relative proportion (%) in parenthesis) collected in maize plants in cultivated fields during the subsequent cropping seasons in two different localities in the central region of Kenya in 2017 and 2018.

Localities	Total Number	Stemborer Species Composition		
		*Busseola. fusca*	*Sesamia calamistis*	*Chilo partellus*
Makutano	515	-	230 (44.66)	285 (55.34)
Murang’a	685	503 (73.43)	182 (26.57)	-
Total Number	1200	503 (41.92)	412 (34.33)	285 (23.75)

-: species was absent.

**Table 7:** The Morista–Horn similarity index between maize plants versus maize residues and between maize plants versus wild plants in the carry-over of lepidopteran maize stemborers and associated larval/pupal parasitoid species.

Localities	Morista–Horn Index (C*mH*)			
	Lepidopteran Maize Stemborer Species		Associated Larval/Pupal Parasitoid Species	
	Maize Plants vs. Maize Residues	Maize Plants vs. Wild Plants	Maize Plants vs. Maize Residues	Maize Plants vs. Wild Plants
Makutano	0.96	0.37	0.99	0.30
Murang’a	0.97	0.31	0.99	0.02

A value close to 1 indicates a greater similarity between the two habitats and vice versa.

**Table 8:** The parasitism rate of *Cotesia flavipes* and *Cotesia sesamia* after and during the release according to the stemborer species and refugia habitat.

Parasitoids	Stemborer Species	Parasitism Rates (%)			Proportion Test		
		Maize Plants	Maize Residues	Wild Plants	χ^2^	*df*	*p*
*Cotesia flavipes* (In Makutano)	*Sesamia calamistis*	3.48 a	5.68 a	5.00 a	0.8	2	0.7
	*Chilo partellus*	2.11 a	1.49 a	0.00 a	0.5	2	0.8
*Cotesia sesamia* (In Murang’a)	*Sesamia calamistis*	2.39 a	3.10 a	0.00 a	0.4	2	0.8
	*Busseola fusca*	3.85 a	3.65 a	0.00 a	0.4	2	0.8

The means in the same row followed by the same letters did not differ significantly.

## Supplementary Materials

The following are available online at https://www.mdpi.com/2075-4450/10/7/191/s1, Figure S1: Number of stemborers of *Busseola fusca*, *Sesamia calamistis* and *Chilo partellus* found in maize residues and wild plants per 100 maize residues or wild plants sampled in two agro-ecological zones (Kitale & Mtito Andei and Kakamega & Muhaka) during non-cropping seasons in 2006 and 2007. Significant differences at 5% level between maize residues vs. wild plants are shown by different letters determined using Tukey’s multiple comparisons tests with the R package “lsmeans”, following generalized linear model (GLM) with negative binomial error distribution. Figure S2: Principal component biplot showing the relation between abundance of stemborer species in maize plants of cultivated fields during cropping season and those in either maize residues or wild plant during non-cropping season in two agro-ecological zones of Kenya (Kitale & Mtito Andei and Kakamega & Muhaka) during non-cropping seasons. Table S1: Stemborer species composition and total abundance in maize residues and wild plants during non-cropping seasons in different agro-ecological zones in Kenya (from Mailafiya [12]). Table S2: Parasitoids composition and abundance from stemborers species in maize residues and wild plants during non-cropping seasons in different agro-ecological zones in Kenya (from Mailafiya [12]). Table S3: Morista-Horn similarity index between maize fields vs. maize residues and between maize fields vs. wild plants in the carry-over of lepidopteran maize stemborer species. Table S4: Morista-Horn similarity index between maize fields vs. maize residues and between maize fields vs. wild plants in the carry-over of lepidopteran maize stemborers associated larval and pupal parasitoids species.
