# Supplementary material for: Carry-Over Niches for Lepidopteran Maize Stemborers and Associated Parasitoids during Non-Cropping Season
Source: Insects. 2019 Jun 28;10(7):191. doi: 10.3390/insects10070191 (PMC6681391; doi:10.3390/insects10070191)
Supplement: Supplementary file 1 [file insects-10-00191-s001.pdf]

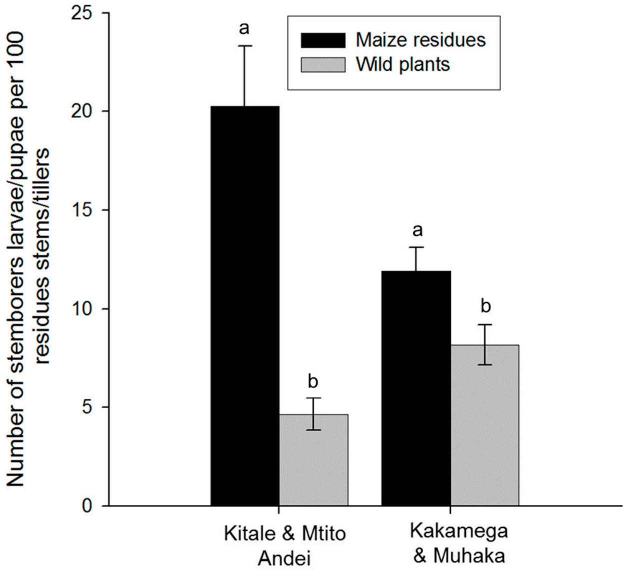

Figure S1. Number of stemborers of *Busseola fusca*, *Sesamia calamistis* and *Chilo partellus* found in maize residues and wild plants per 100 maize residues or wild plants sampled in two agro-ecological zones (Kitale & Mtito Andei and Kakamega & Muhaka) during non-cropping seasons in 2009 and 2009. Significant differences at 5% level between maize residues vs. wild plants are shown by different letters determined using Tukey's multiple comparisons tests with the R package "lsmeans", following generalized linear model (GLM) with negative binomial error distribution.

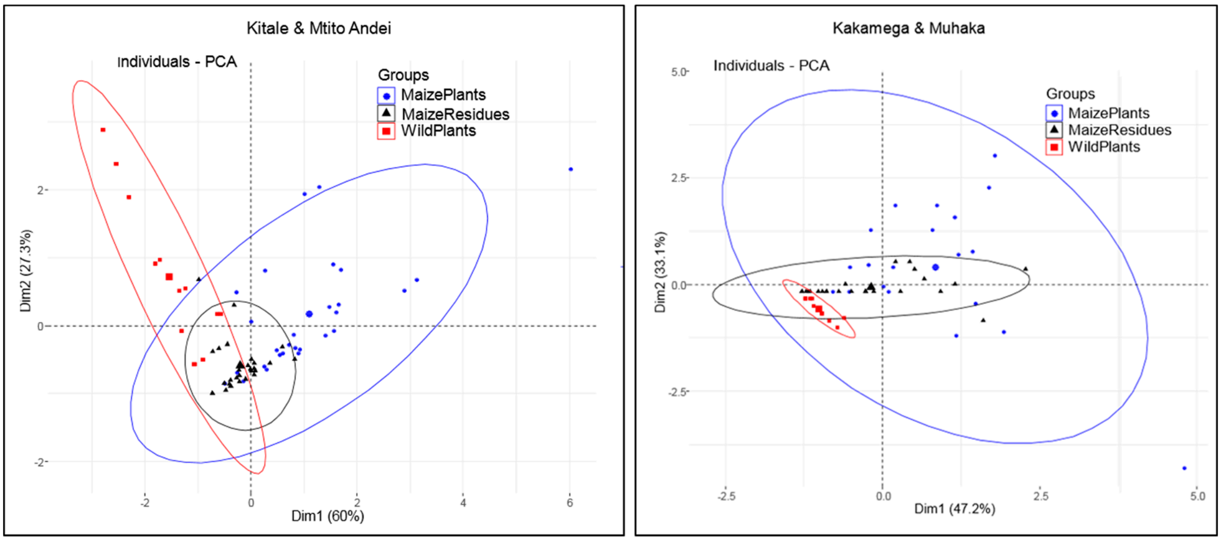

Figure S2. Principal component biplot showing the relation between abundance of stemborer species in maize plants of cultivated fields during cropping season and those in either maize residues or wild plant during non-cropping season in two agro-ecological zones of Kenya (Kitale & Mtito Andei and Kakamega & Muhaka) during non-cropping seasons.

Table S1. Stemborer species composition and total abundance in maize residues and wild plants during non-cropping seasons in different agro-ecological zones in Kenya (from Mailafiya [12]).

Habitats	Total Number	Stemborer Species Composition					
		Bf	Sc	Cp	Co	Bse	Os
		Kitale & Mtito Andei					
Maize residues	1090	641	180	197	49	17	6 Es
Wild plants	146	6	4	2	7	86	41
Sorghum arundinaceum *	18	4	3	2	-	9	-
Panicum maximum *	29	2	-	-	-	26	1 Mn
Panicum sp. *	9	-	-	-	-	2	5 Ppi, 2 Mn
Eleusine jaegen *	34	-	-	-	2	30	2 Ppi
Cynodon dactylon *	17	-	1	-	4	-	7 Ssp, 5 Csp
Cyperus sp. †	2	-	-	-	-	-	2 Sv
Echinochloa haploclada *	20	-	-	-	1	7	12 Mni
Rottboellia cochinchinensis *	17	-	-	-	-	12	5 Esp
		Kakamega & Muhaka					
Maize residues	453	126	141	138	27	15	6 Sp
Wild grasses	338	5	13	11	81	125	103
Panicum maximum *	107	-	6	2	76	6	17 Sp
Panicum sp. *	80	-	3	3	-	55	14 Csp, 5 Mn
Cyperus sp. †	33	-	-	-	-	9	24 Sno
Sorghum arundinaceum *	12	-	3	6	1	-	1 Ppi, 1 Es
Pennisetum trachyphyllum *	6	2	-	-	-	-	4 Sn
Pennisetum purpureum *	26	3	-	-	-	18	5 Ppi
Cyperus dereilema †	4	-	-	-	-	-	4 Ppi
Cynodon sp. *	3	-	-	-	-	-	2 Ppi, 1 Scs
Scleria racemosa †	39	-	-	-	-	35	1 Sv, 3 Mn
Chloris gayana *	5	-	-	-	-	-	5 Mn
Setaria megaphylla *	4	-	-	-	-	2	2 Bs
Cynodon dactylon *	19	-	1	-	4	-	8 Ssp, 6 Csp

Os = Other species. Noctuidae (Sn = *Sciomesa nyei*; Ppi = *Pirateolea piscator*; Sv = *Sciomesa venata*; Ssp = *Sciomesa* sp nov; Mn = *Manga nubifera*; Sp = *Sesamia poephaga*; Sno = *Sesamia nonagriodes*; Sc = *Sesamia calamistis*; Bse = *Busseola segeta*; Bs = *Busseola sensu*; Bf = *Busseola fusca*) / Crambidae (Co = *Chilo orichalcociliellus*; Cp = *Chilo partellus*; Csp = *Chilo* sp) / Pyralidae (Esp = *Ematheudes* sp.; Es = *Eldana saccharina*; Mni = *Mussidia nigrivenella*; Scs = *Schoenobius* sp). Wild grasses: * Poaceae, † Cyperaceae.

Table S2. Parasitoids composition and abundance from stemborers species in maize residues and wild plants during non-cropping seasons in different agro-ecological zones in Kenya (from Mailafiya [12]). .

Parasitoid Species	Borer Species	Borer Stages	Wild Plants	Total Number	Kitale & Mtito Andei		Kakamega & Muhaka	
					Maize Residues	Wild Plants	Maize Residues	Wild Plants
Hymenoptera: Braconidae								
Cotesia flavipes	Cp, Sc	larva	Sa	22	-	-	17	5
Cotesia sesamiae	Bf, Sc	larva	Sa	13	9	4	-	-
Dolichogenidea polaskezi	Bf	larva	-	1	1	-	-	-
Macrocentrus sp.	Tr	larva	Cr	1	-	-	-	1
Hymenoptera: Ichneumonidae								
Syzectus sp.	Co, Cp,	pupa	Sa, Pm	5	-	2	-	3
Amouramorpha sp.	Tr	larva	Cr	1	-	1	-	-
Hymenoptera: Eulophidae								
Pediobius furvus	Cp, Sc	pupa	Sa	3	-	-	3	-
Diptera: Muscidae								
Atherigona sp	Cp	larva	Pm	2	-	-	-	2
Diptera: Chloropidae								
Chloropid sp	Bf	larva	Sm	2	-	2	-	-
Diptera: Tachinidae								
Sturmiopsis parasitica	Bf	larva	Sa	2	1	1	-	-
Siphona (Meigen) *sp.*	Bf, Mn	larva	Pm	2	2	-	-	-

Borer species: Bf = *Busseola fusca*, Sc = *Sesamia calamistis*, Cp = *Chilo partellus*, Co = *Chilo orichalcocillielus*, Tr = Tortricidae, Mn = *Manga nubifera*; Plant species: Sa = *Sorghum arundinaceum*, Pm = *Panicum maximum*, Cr = *Cyperus rotundus*, Sm = *Setaria megaphylla*. No. = Total number of parasitized larvae recorded for a given parasitoid species, - absent.

Table S3. Morista-Horn similarity index between maize fields vs. maize residues and between maize fields vs. wild plants in the carry-over of lepidopteran maize stemborer species.

Agro-Ecological Zones	Morista-Horn Index (C*mH*)	
	**Maize Fields vs. Maize Residues**	**Maize Fields vs. Wild Plants**
Kitale & Mtito Andei	0.95	0.04
Kakamega & Muhaka	0.99	0.14

The value close to 1 indicates a greater similarity between the two habitats and vis versa.

Table S4. Morista-Horn similarity index between maize fields vs. maize residues and between maize fields vs. wild plants in the carry-over of lepidopteran maize stemborers associated larval and pupal parasitoids species.

Agro-Ecological Zones	Morista-Horn Index (C*mH*)	
	**Maize Fields vs. Maize Residues**	**Maize Fields vs. Wild Plants**
Kitale & Mtito Andei	0.98	0.68
Kakamega & Muhaka	0.97	0.63

The value close to 1 indicates a greater similarity between the two habitats and vis versa.
